# Combination of pharmacotherapy and psychotherapy in the treatment of chronic depression: A systematic review and meta-analysis

**DOI:** 10.1186/1471-244X-12-61

**Published:** 2012-06-13

**Authors:** Alessa von Wolff, Lars P Hölzel, Annika Westphal, Martin Härter, Levente Kriston

**Affiliations:** 1 Department of Medical Psychology, University Medical Center Hamburg-Eppendorf, Hamburg, Germany; 2 Department of Psychiatry and Psychotherapy, University Medical Center Freiburg, Freiburg, Germany

**Keywords:** Chronic depression, Psychotherapy, Pharmacotherapy, Meta-analysis, Systematic review

## Abstract

**Background:**

Chronic depression represents a substantial portion of depressive disorders and is associated with severe consequences. This review examined whether the combination of pharmacological treatments and psychotherapy is associated with higher effectiveness than pharmacotherapy alone *via* meta-analysis; and identified possible treatment effect modifiers *via* meta-regression-analysis.

**Methods:**

A systematic search was conducted in the following databases: Cochrane Central Register of Controlled Trials (CENTRAL), MEDLINE, EMBASE, ISI Web of Science, BIOSIS, PsycINFO, and CINAHL. Primary efficacy outcome was a response to treatment; primary acceptance outcome was dropping out of the study. Only randomized controlled trials were considered.

**Results:**

We identified 8 studies with a total of 9 relevant comparisons. Our analysis revealed small, but statistically not significant effects of combined therapies on outcomes directly related to depression (BR = 1.20) with substantial heterogeneity between studies (I² = 67%). Three treatment effect modifiers were identified: target disorders, the type of psychotherapy and the type of pharmacotherapy. Small but statistically significant effects of combined therapies on quality of life (SMD = 0.18) were revealed. No differences in acceptance rates and the long-term effects between combined treatments and pure pharmacological interventions were observed.

**Conclusions:**

This systematic review could not provide clear evidence for the combination of pharmacotherapy and psychotherapy. However, due to the small amount of primary studies further research is needed for a conclusive decision.

## Background

Approximately 20% of all patients who experience a major depressive episode develop a chronic course [1] and approximately 47% of patients who are treated in mental health care facility suffer from some form of chronic depression [2]. Four subtypes of chronic depression are usually distinguished: (1) dysthymia, (2) chronic major depression, (3) recurrent major depression with incomplete remission between episodes, and (4) double depression [3]. Dysthymic disorder is defined as a mild condition that is chronic and persistent for at least 2 years. Major depressive episode, chronic type, refers to a more severe condition that meets full criteria for major depression continuously for a minimum of 2 years. Patients who have recovered to the point where they no longer meet full criteria for a major depressive episode but continue to experience significant symptoms for a total duration of illness greater than 2 years are referred to as recurrent major depression with incomplete remission during episodes. The superimposition of a major depressive episode on antecedent dysthymia is referred to as double depression [3].

Chronic depression is associated with increased functional impairment [4], increased health care utilization, and higher rates of hospitalization compared with non-chronic forms of depression [1,5].

An increasing number of studies have assessed the effectiveness of several pharmacological, psychotherapeutic, and combined pharmacological and psychotherapeutic interventions for the treatment of chronic depression in the last several decades. Several meta-analyses have confirmed the effectiveness of pharmacological treatments [6,7] and systematic reviews have highlighted the effectiveness of psychotherapy in the treatment of chronically depressed patients [8,9].

Because different effective interventions are available for the treatment of chronic depression, a comparative analysis of the effectiveness of these different interventions is of great clinical interest. Although, both systematic reviews and current treatment guidelines recommend combined psychotherapeutic and pharmacological interventions for the treatment of chronic depression, these recommendations are based on a limited amount of evidence [8-11].

Psychotherapy continues to face numerous barriers, such as limited access in underserved areas and higher short-term costs when delivered by mental health care professionals [12]. Therefore, the delineation of the conditions under which combined treatment provides significantly greater effectiveness than pure pharmacological intervention is of high relevance. However, whether all patients receive equal benefit from the addition of psychotherapy to a pharmacological intervention is not known. A recent meta-analysis reported that the efficacy of psychotherapeutic interventions increases with the number of sessions and patients who suffer from dysthymia receive less benefit from combined treatment compared to pharmacotherapy alone than patients in other diagnostic subgroups [9].

Because new data on the comparative effectiveness of combined interventions *versus* pharmacotherapy alone are available from a large randomized controlled trial [13], we decided to summarize empirical evidence on the effectiveness of combined psychotherapeutic and pharmacological treatments for chronic depression compared to pharmacotherapy alone by means of a systematic review. Our aim was to especially focus on outcomes that are relevant for clinicians who treat patients with chronic depression, such as the response to treatment, remission, quality of life, and acceptance of treatments (dropout), and to further examine whether the addition of psychotherapy to a pharmacological intervention is more effective in certain subgroups of patients (*e.g.* according to diagnosis).

The objectives of this systematic review are to examine whether the augmentation of pharmacological treatments with psychotherapy is associated with higher effectiveness compared to pharmacotherapy alone and identify possible treatment effect modifiers, *i.e.* factors that may influence the size of effects of augmentation of pharmacological treatments with psychotherapy.

## Methods

The methods and results are reported in accordance with the Preferred Reporting Items for Systematic reviews and Meta-Analysis (PRISMA) statement [14]. Methods were specified *a priori* in a freely accessible review protocol that included a detailed description of the methods, which are summarized briefly here [15].

### Eligibility criteria

Studies that were conducted in adults with a diagnosis of chronic major depression, dysthymia, double depression, or recurrent depression without complete remission between episodes were included. The diagnosis of depression had to rely on a formal classification system. Studies focusing on preselected samples (*e.g.* predefined comorbidities) of chronically depressed patients were excluded.

Combined psychotherapeutic and pharmacological interventions that focused primarily on the treatment of depressive symptoms were considered. Only acute treatments (no maintenance or continuation treatments) were included. Psychotherapeutic interventions had to fulfill the following criteria: 1) the intervention must be based on a scientific theory; 2) a minimum of one contact between therapist and patient must take place; and 3) the intervention must consider the personal needs of the patient and must be individually tailored in an interpersonal process. Combined interventions included the administration of one or more antidepressant pharmacological agents combined with one or more psychotherapeutic interventions. Somatic, non-pharmacological, and organizational interventions were not considered.

The comparator treatment needed to be an antidepressant pharmacological intervention alone.

Only studies that reported at least one outcome to assess the efficacy of the interventions and only randomized controlled trials (RCTs) were included.

### Search strategy

An electronic database search was conducted in the following databases on January 18, 2010: Cochrane Central Register of Controlled Trials (CENTRAL), MEDLINE, EMBASE, ISI Web of Science, BIOSIS, PsycINFO, and CINAHL. A disease component was combined (AND) with a design component for all searches: *(((chron$ adj3 depress$) or dysthym$ or (double adj1 depress$) or (treatment adj1 resist$ adj1 depress$) or (non adj1 respon$ adj3 depress$) or (recurrent adj3 depress$)).ab,ti,sh.) AND ((random$ or rct).ab,ti. or random$.sh.)* (*e.g.*, MEDLINE). No language restrictions were applied and all publications from 1970 forward were considered.

Additionally, all volumes of the Archives of General Psychiatry, the Journal of Consulting and Clinical Psychology, and the Journal of Affective Disorders were searched by hand beginning with the year 1970. The reference lists of all included studies and relevant reviews were searched and a cited reference search in the Social Sciences and Science Citation Index was performed for all included studies. The first author of all included studies was contacted for further information regarding published and unpublished trials.

### Study selection

Two reviewers independently screened title and abstract of all identified articles. Next two reviewers independently examined the full texts of all potentially relevant studies using inclusion criteria that were *a priori* defined. Disagreement was resolved by discussion.

### Data collection and assessment of methodological quality

A data extraction form of study characteristics, treatment characteristics, sample characteristics and outcomes was developed.

Risk of bias was assessed in accordance with the Cochrane Collaboration’s Risk of Bias tool [16]. Two researchers independently conducted the data extraction of outcome data and the assessment of risk of bias. Disagreements were resolved by discussion. The absolute proportion of corresponding judgments and Cohen's kappa coefficient were calculated to assess inter-rater agreement.

### Data synthesis

The primary efficacy outcome was the response to treatment, which was defined as an at least 50% decrease on a depression scale from baseline to the end of treatment. Secondary efficacy outcomes included metric outcomes of depression scales at the end of intervention and at follow-up, the dichotomous outcome remission, and metric outcomes on any quality of life scale. The primary acceptance outcome was dropping out of the study due to any reason. The primary endpoint was the end of intervention for all outcomes irrespective of intervention duration. Follow-up was defined as at least 6 months after the end of intervention.

The statistical analysis followed actual guidelines [16-18]. Effectiveness measures were benefit ratios (BR; a ‘risk ratio’ for positive endpoints) for dichotomous effectiveness outcomes and odds ratios for dropout rates with corresponding 95% confidence intervals (CI). Odds ratios were chosen because we expected dropout rates to be a rare outcome with highly varying baseline rates. Standardized mean differences (SMD) were calculated for continuous measures.

Effect sizes were calculated using the intention-to-treat principle for all studies. All randomized patients were included in the analyses of primary outcomes irrespective of how the authors of the primary studies defined their intention-to-treat sample. For secondary outcomes the definition of the intention-to-treat sample provided by the authors was followed. In all analyses a random effects model with inverse variance weights were applied [19].

Statistical heterogeneity between study results was tested for significance using Cochran’s Q-test and quantified using the I² statistic [20].

Possible publication bias was investigated using visual examination of funnel plots and applying Egger’s test [21].

### Meta-regression analysis

Heterogeneity among primary study findings was explored using meta-regression analyses that examined a series of possible effect modifiers. *A priori* defined analyses were performed according to the type of psychotherapeutic intervention (brief supportive psychotherapy (BSP), cognitive behavioural therapy (CBT), Cognitive Behavioural Analysis System of Psychotherapy (CBASP), Interpersonal Psychotherapy (IPT)), the subtype of chronic depression (percentage patients with dysthymia, double depression, chronic major depression, recurrent depression without complete remission between episodes in the sample), the onset and severity of the target disorder (percentage of patients who reported an early onset of the disorder and baseline severity of depression), and study quality (high quality *vs.* medium or low quality) [15]. Additional *a posteriori* analyses were performed according to the total number of patients included, setting (outpatient *vs.* inpatient), the number of sessions, treatment duration (in weeks), the use of selective serotonin reuptake inhibitors (SSRIs) as antidepressant medication, mean age of the sample, and the percentage of females in the sample.

Inverse variance-weighted meta-regression models were used to test possible effect modifiers formally [22-24]. Random effects models were estimated *via* the restricted information maximum likelihood procedure. We chose to conduct meta-regression analysis instead of subgroup analysis for categorical effect modifiers because this method allows applying a random effects model and provides a consistent framework for all analyses (metric as well as categorical effect modifiers). All analyses were conducted for two types of outcome measures: benefit ratios for treatment response and standardized mean differences for depressive symptoms at end of intervention. Bivariate association of treatment effect modifiers were examined to reveal possible multicollinearity.

In order to examine the influence of single studies on the findings leave-one-out sensitivity analyses were conducted for the primary outcomes response and dropout. Additional leave-one-out sensitivity analyses were performed for the meta-regression-analyses.

Analyses were performed using Review Manager 5 (The Nordic Cochrane Centre, The Cochrane Collaboration, Copenhagen) and PASW Statistics 18 (SPSS, Inc., Chicago, IL) with an additional macro by Wilson [25].

## Results

### Study selection

**Figure 1:**
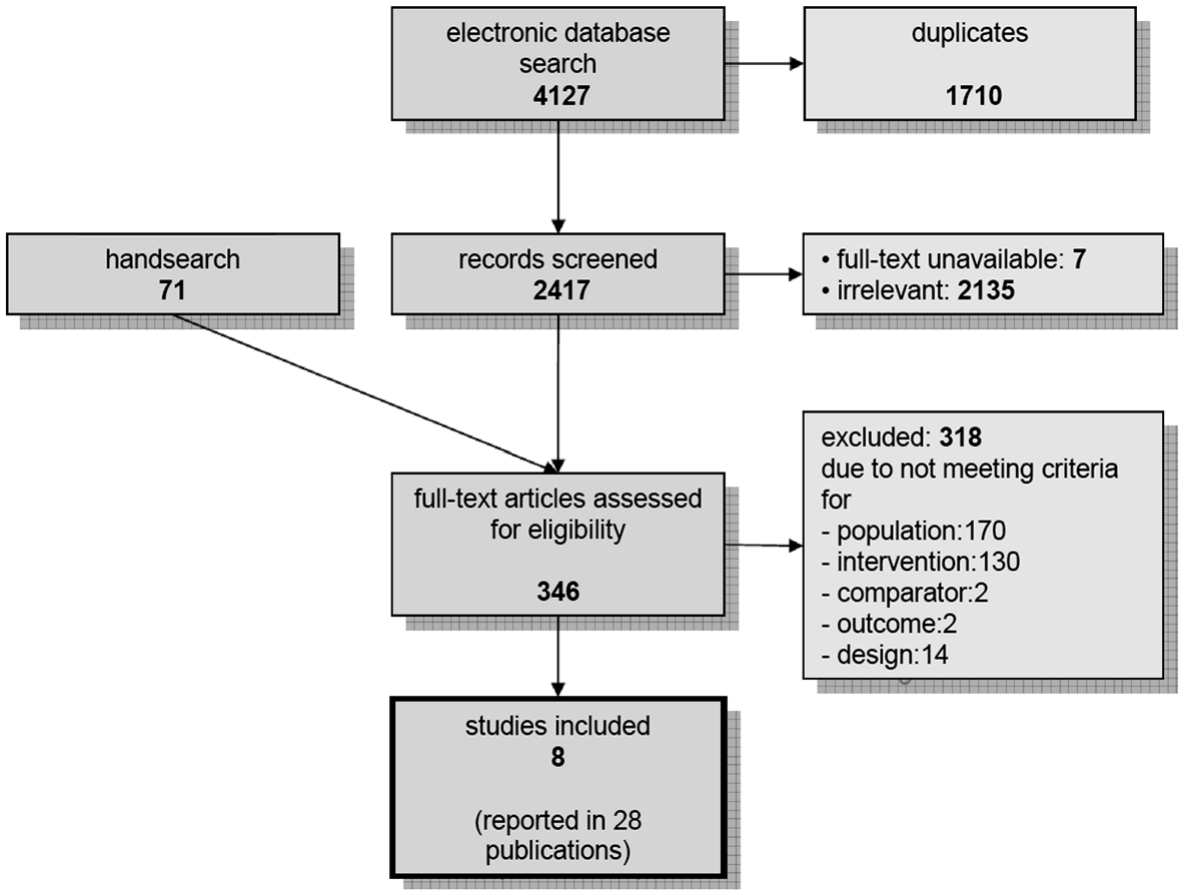
Study flow diagram.

After the removal of 1,710 duplicates 2,417 potentially relevant publications were identified through the electronic database research (Figure 1). The number of publications was reduced to 304 through screening of title and abstract. The hand searches identified another 49 possibly relevant studies. Of these 353 studies, seven studies were not available. The full texts of the available studies were screened and eight primary studies [13,26-32] reported in 28 publications with a total of 1,618 participants were included in the systematic review. The included studies comprise a total of nine comparisons of a combined intervention *versus* pharmacotherapy alone.

### Study characteristics

The sample sizes ranged from N = 26 to N = 476. Most studies (5/8) used SSRIs as the pharmacological intervention. IPT was used as the psychotherapeutic intervention in four studies, CBASP and CBT were each used in two studies and one study used BSP. Half of the studies (4/8) were conducted in the United States. Most studies focused on samples with a majority of dysthymic patients. The average age of the study participants ranged from 37 to 45 years. All studies included a majority of women. The detailed characteristics of each study are presented in Table 1.

**Table 1:** Study characteristics

	**Psychotherapy**	**Medication**	**Diagnosis**	**Mean age of sample**	**% female in sample**	**N se**	**Treatment duration (weeks)**	**N rand**	**Country**
Browne 2002[26]	IPT	Sertraline (SSRI)	Dys, DD	42.2	68	12	24	476	Canada
deMello 2001[27]	IPT	Moclobemide	Dys, DD	38.8	80	16	32	35	Brazil
Keller 2000[28]	CBASP	Nefazodone	cMD, rec, DD	43.0	65	16	12	453	USA
Kocsis 2009[13]	CBASP/BSP	SSRIs	cMD, rec, DD	45.4	55	16/18	12	491	USA
Markowitz 2005[29]	IPT	Sertraline (SSRI)	Dys	42.3	63	16	16	45	USA
Miller 1999[30]	CBT	Amitriptyline/Desipramine	Dys	37.4	81	40	20	26	USA
Ravindran 1999[31]	CBT	Sertraline (SSRI)	Dys	38.0	58	12	12	47	Canada
Schramm 2008[32]	IPT	Sertraline (SSRI)/Amitriptyline	cMD, DD	42.8	67	15	5	45	Germany

N se = number of psychotherapy sessions; N rand = patients randomized to relevant study arms; IPT = Interpersonal Psychotherapy; CBASP = Cognitive Behavioural Analysis System of Psychotherapy; BSP = Brief Supportive Psychotherapy; CBT = Cognitive Behavioural Therapy; Dys = dysthymia; DD = double depression; cMD = chronic major depression; rec = recurrent depression without complete remission between episodes.

### Risk of bias within studies

The overall risk of bias was evaluated as “low” for five studies and as “medium” for two studies. Only one study was judged to have a “high” risk of bias. Knowledge of allocation was adequately concealed in nearly all studies (7/8), whereas an adequate concealment of the allocation was reported in only four of the studies. The results of the methodological quality assessment are presented for individual studies in Table 2. Inter-rater agreement for single quality assessment items ranged from 4/8 to 8/8 (kappa coefficients ranged from -.33 to 1.0).

**Table 2:** Risk of bias in individual studies

	**1**	**2**	**3**	**4**	**5**	**6**	**Global Judgment**
Browne 2002	yes	yes	yes	yes	yes	no	low
deMello 2001	unclear	unclear	yes	unclear	yes	no	medium
Keller 2000	yes	yes	yes	yes	yes	yes	low
Kocsis 2009	yes	yes	yes	yes	no	yes	low
Markowitz 2005	yes	unclear	yes	yes	yes	yes	low
Miller 1999	no	yes	unclear	unclear	no	no	high
Ravindran 1999	yes	unclear	yes	yes	no	yes	medium
Schramm 2008	yes	unclear	yes	yes	yes	yes	low
Summary “yes”	6/8	4/8	7/8	6/8	5/8	5/8	4/8

1: allocation sequence adequately generated; 2: allocation adequately concealed; 3: knowledge of allocation adequately prevented; 4: incomplete outcome adequately addressed; 5: free of selective outcome reporting; 6: free of other problems.

### Synthesis of results

One study reported two intervention arms with a combined treatment and one arm with a pharmacological treatment and thus provided two comparisons of interest for the current meta-analysis [13]. We cut the number of patients in the pharmacological arm in half for all analyses to avoid including patients more than once in the analysis.

The results of one study [30] proved to be an outlier in the analysis of depressive symptoms at the end of treatment; the effect size estimate differed strongly and implausibly from the other studies (SMD approximately ten times higher than any other estimate). This same study was judged to have a high risk of bias. Therefore, this study was excluded from all further analysis.

**Figure 2:**
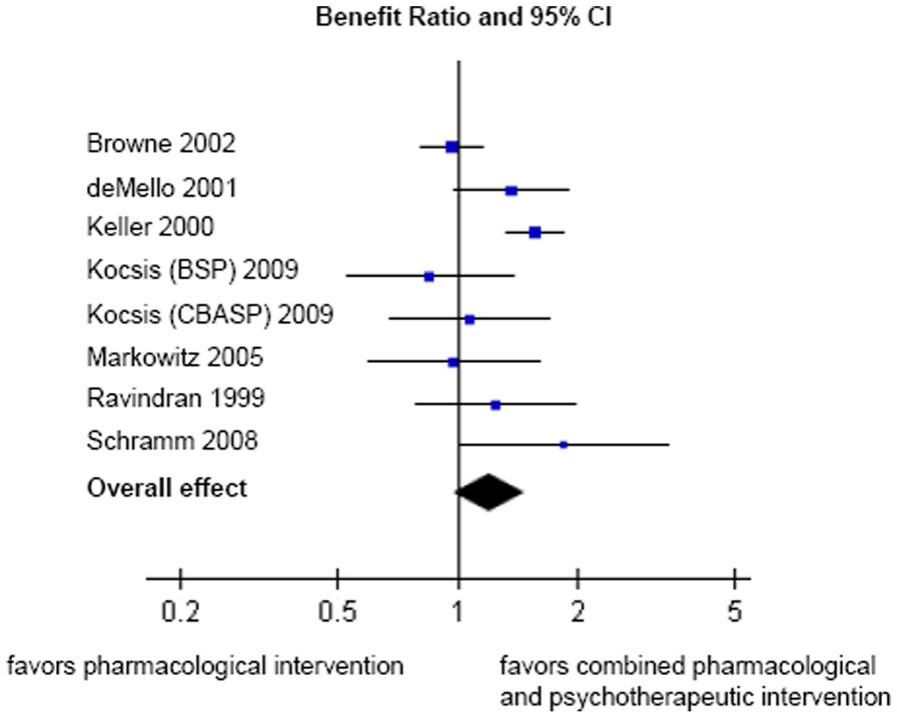
Forest plot (response).

No statistically significant difference (at p < 0.05) in response rates between combined treatments and pharmacological interventions alone were found. However, combined treatments tended to be superior to pharmacological therapies (BR = 1.20; 95% CI: 0.98-1.48; p = 0.08). This result corresponds with a number needed to treat (NNT) of 20. Leave-one-out sensitivity analysis showed the greatest increase of the overall effect (BR = 1.29; p = 0.01) after removal of the study by Browne and colleagues [26] and the greatest decrease (BR = 1.10; p = 0.25) after removal of the study by Keller and colleagues [28]. A forest plot of this comparison is displayed in Figure 2. Accordingly, the results for rates of remission (BR = 1.25; 95% CI: 0.97-1.61; p = 0.08; NNT = 50) and depressive symptoms at end of intervention (SMD = 0.13; 95% CI: -0.08-0.34; p = 0.22) indicated a possible superiority of combined treatments compared to pharmacological treatments alone. Again, none of these comparisons reached statistical significance at p < 0.05.

Substantial statistical heterogeneity among the results of the primary studies was observed for all outcomes (I² = 67% for response; I² = 52% for remission; I² = 50% for depressive symptoms).

Combined treatment showed statistically significantly better quality of life outcomes than pure pharmacological interventions at the end of active treatment (SMD = 0.18; 95% CI: 0.07-0.29; p = 0.002).

Dropout rates did not differ statistically between combined treatments and pharmacological interventions alone (OR = 0.84; 95% CI: 0.64-1.11; p = 0.21). These results were not heterogeneous across studies (I² = 0%). Leave-one-out sensitivity analysis showed the greatest increase of the overall effect (OR = 0.90; p = 0.56) after removal of the study by Keller and colleagues [28] and the greatest decrease (OR = 0.79; p = 0.15) after removal of the study by Browne and colleagues [26].

Four studies provided data of depressive symptoms at follow-up. Long-term effectiveness at an average of 12.5 months after end of active treatment was not statistically significantly different between combined and pharmacological treatments (SMD = 0.25, 95% CI: -0.15-0.66; p = 0.22).

Results for each individual study concerning each outcome as well as overall effects are displayed in Table 3.

**Table 3:** Results of all outcomes (individual studies and overall effect)

	**Response**	**Remission**	**Depressive symptoms**		**Quality of life**^**3**^	**Dropout**
	**BR (95% CI)**	**BR (95% CI)**	**short term**^**1**^	**long term**^**2**^	**SMD (95% CI)**	**OR (95% CI)**
			**SMD (95% CI)**			
Browne 2002	0.97 (0.81,1.16)	0.94 (0.71,1.25)	−0.07 (−0.27,0.13)	0.04 (−0.16,0.24)	0.03 (−0.17,0.23)	0.98 (0.59,1.64)
deMello 2001	1.37 (0.98,1.91)	1.45 (0.81,2.59)	0.48 (−0.34,1.30)	0.50 (−0.38,1.38)	0.65 (−0.17,1.47)	0.44 (0.11,1.70)
Keller 2000	1.56 (1.33,1.84)	2.07 (1.46,2.95)	0.54 (0.19,0.89)	not reported	0.29 (0.11,0.47)	0.76 (0.49,1.17)
Kocsis (CBASP) 2009	1.07 (0.67,1.70)	1.11 (0.67,1.84)	0.12 (−0.23,0.47)	not reported	0.22 (−0.13,0.57)	0.71 (0.30,1.70)
Kocsis (BSP) 2009	0.85 (0.53,1.38)	0.91 (0.54,1.54)	−0.06 (−0.41,0.29)	not reported	0.07 (−0.28,0.42)	0.80 (0.34,1.90)
Markowitz 2005	0.98 (0.59,1.62)	1.26 (0.67,2.35)	−0.27 (−0.86,0.32)	not reported	0.22 (−0.37,0.81)	0.89 (0.21,3.88)
Miller 1999	11.27 (0.70,181.41)	1.29 (0.47,3.51)	4.15 (2.78,5.52)	0.10 (−0.66,0.86)	1.15 (0.33,1.97)	1.36 (0.19,9.91)
Ravindran 1999	1.25 (0.78,1.99)	1.06 (0.57,1.95)	0.05 (−0.54,0.64)	not reported	0.15 (−0.54,0.84)	not estimable
Schramm 2008	1.86 (1.02,3.40)	1.75 (0.80,3.84)	0.53 (−0.06,1.12)	0.63 (−0.06,1.32)	not reported	3.17 (0.56,17.78)
total						
overall effect *	1.20 (0.98,1.48)	1.25 (0.97,1.61)	0.13 (−0.08,0.34)	0.25 (−0.15,0.66)	0.18 (0.07,0.29)	0.84 (0.64,1.11)
p for overall effect *	0.080	0.080	0.220	0.220	0.002	0.210
I² *	67%	52%	50%	42%	0%	0%
p for heterogeneity *	0.004	0.040	0.050	0.180	0.490	0.690

^1^ = symptoms at end of intervention; ^2^ = symptoms at follow-up (follow-up ranged from 8 to 18 months after the end of intervention); ^3^ = in the case of three studies data are based on the Social Adjustment Scale; CI = confidence interval; SMD = standardized mean difference; BR = benefit ratio; OR = odds ratio; CBASP = Cognitive Behavioural Analysis System of Psychotherapy; BSP = Brief Supportive Psychotherapy; p = level of significance; I² = measure of heterogeneity; * results after removal of the study by Miller 1999.

### Risk of bias across studies

**Figure 3:**
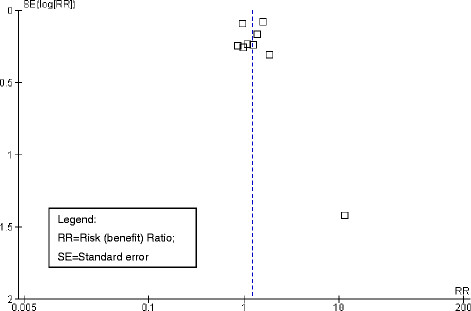
Funnel plot.

With the exception of one study that was previously judged as having a high risk of bias and implausibly large effects [30], no indication of publication bias for the primary outcome *response to treatment* by visual examination of the funnel plot was observed (see Figure 3). Accordingly, no indication for publication bias was found when applying Egger’s test (beta = 0.01; p = 0.98).

### Meta-regression analysis

As mentioned above substantial statistical heterogeneity between studies was observed for the primary outcome response to treatment and depressive symptoms at the end of intervention. These findings indicate relevant variation in effect sizes between studies. Therefore, further exploration of this heterogeneity using meta-regression analysis was conducted.

The results of the meta-regression analysis are presented in Table 4. For both outcomes *response to treatment* as well as *depressive symptoms at end of interventions* three relevant treatment effect modifiers were identified. First, combined treatment yielded significantly higher effects in studies of patients with chronic major depression compared to studies of patients who suffer from dysthymia (p = 0.028). This result did not reach statistical significance for the outcome *depressive symptoms* at end of intervention (p = 0.252), but it accounted for nearly 70% of the effect variance across studies (as suggested by the R-squared estimate). Expressed in differences of benefit ratios this result indicates, that in a study with only dysthymic patients the estimated benefit risk for response between a combined treatment and a pure pharmacological treatment would be 0.95, whereas in a study with patients suffering from chronic major depression the estimated benefit ratio would be 4.02. Leave-one-out sensitivity analysis revealed that the diagnostic subgroup did not serve as a statistically significant treatment effect moderator when the study by Keller and colleagues [28] or the study by deMello and colleagues [27] were removed from the analysis.

**Table 4:** Results of meta-regression analysis

	**Response (lnBR)**				**Depression symptoms (SMD)**			
	**constant**^1^	**beta**^2^	**p**	**R²**	**constant**^1^	**beta**^2^	**p**	**R²**
% early onset	0.34	−0.30	.564	.06	0.43	−0.63	.255	.19
baseline severity	−0.66	0.04	.160	.28	−0.89	0.05	.129	.32
diagnosis (reference dysthymia)	−0.05			.69	−0.18			.69
% chronic MD		1.44	.028			0.88	.252	
% recurrent without remission		−1.16	.211			−0.88	.312	
% double depression		0.40	.085			0.81	.111	
mean age	1.46	−0.03	.463	.09	0.75	−0.02	.760	.01
% female	−0.55	1.13	.373	.13	−0.77	1.43	.372	.12
sessions	−0.13	0.02	.631	.03	−0.27	0.03	.560	.04
treatment duration (weeks)	0.31	−0.01	.521	.06	0.37	−0.02	.137	.19
setting outpatient (*vs.* inpatient)	0.39	−0.24	.348	.15	0.29	−0.20	.469	.07
type of PT (reference IPT)	0.07			.57	−0.01			.40
CBT		0.15	.555			0.06	.846	
CBASP		0.33	.002			0.34	.028	
BSP		−0.23	.365			−0.05	.807	
type of medication SSRI (*vs.* others)	0.42	−0.40	≤.001	.72	0.53	−0.54	.003	.64
low risk of bias (*vs.* medium)	0.28	−0.12	.599	.05	0.21	−0.10	.754	.01
total N in study	0.23	−0.00	.756	.02	0.13	0.00	.980	.00
multiple regression analysis	0.33			0.79	0.38			.78
% chronic MD		0.51	.317			0.54	.237	
CBASP		−0.66	.769			−0.01	.969	
type of medication SSRI (*vs.* others)		−0.35	.021			−0.46	.035	

^1^ unstandardized regression constant (equals treatment effect reference category) ^2^ unstandardized regression weight; positive values indicate superiority of the add-on psychotherapy to medication over medication alone; lnBR = natural logarithm of Benefit Risk; SMD = Standardized Mean Difference; p = level of significance; R² = explained variance; MD = Major Depression; PT = psychotherapy; IPT = Interpersonal Psychotherapy; CBT = Cognitive Behavioural Therapy; CBASP = Cognitive Behavioural Analysis System of Psychotherapy; BSP = Brief Supportive Psychotherapy.

Apart from diagnostic subgroups the sample characteristics of the studies such as mean age, percentage of women, or percentage of patients reporting an early onset of their disorder were not associated statistically significantly with differences in effect sizes between combined treatments and pure pharmacological interventions.

Second, in studies that added CBASP to a pharmacological intervention, differences between the combined intervention and the pharmacological intervention were significantly greater than in studies that used IPT (p = 0.002 for response; p = 0.028 for severity of depression). IPT was used as reference intervention because it was the most frequently used form of psychotherapy. This effect corresponds to a benefit ratio of 1.07 for IPT compared to an estimated benefit ratio of 1.49 for CBASP. No effect of the number of psychotherapeutic sessions or the duration of treatment was observed. Leave-one-out sensitivity analysis revealed that the type of psychotherapeutic intervention did not serve as a statistically significant treatment effect moderator when the study by Keller and colleagues [28] or the study by Browne and colleagues [26] were removed from the analysis.

Third, in studies that used SSRIs as the pharmacological intervention, the add-on effect of the psychotherapeutic intervention was diminished compared to studies that used other antidepressant medications (p < 0.001 for response; p = 0.003 for severity of depression). This effect corresponds to an estimated benefit ratio of 1.02 in a study that uses SSRIs s the antidepressant medication (which indicates no differences in the response rates between combined treatments and pure pharmacological treatments) compared to an estimated BR of 1.52 in study with other types of medication. Leave-one-out sensitivity analysis revealed that the type of medication did not serve as a statistically significant treatment effect moderator when the study by Keller and colleagues [28] was removed from the analysis.

The risk of bias of the individual studies and the number of patients that were included in the analysis did not serve as treatment effect modifiers.

We conducted further exploratory multiple regression analyses testing all three relevant effect modifiers (the percent of patients with chronic major depression, the use of CBASP as psychotherapeutic intervention and the use of SSRIs as pharmacological intervention) in one model. The results indicated that SSRIs significantly reduced the effect of combined psychotherapeutic and pharmacological interventions compared to a pure pharmacological intervention (p = 0.021 for response; p = 0.035 for severity of depression). However, the use of CBASP as the psychotherapeutic intervention and the percent of patients with chronic major depression did not influence the differences in effect sizes between combined interventions and pharmacological interventions alone. Inter-correlations between the three treatment effect modifiers were small to moderate and not statistically significant (Spearman’s rho: -0.33 to 0.34). Leave-one-out sensitivity analysis revealed that the type of medication did not serve as a statistically significant treatment effect moderator when the study by deMello [27] was removed for the analysis for the outcome *response to treatment*. No statistically significant effect was found for the outcome *depressive symptoms at end of intervention* after removal of the study by Keller and colleagues [28] or the study by Kocsis and colleagues [13].

## Discussion

### Summary

A small and statistically not significant effect of combined psychotherapeutic and pharmacological interventions on outcomes that are directly related to depression (response, remission, symptom severity) with a substantial variation of effect sizes between studies was revealed. A more detailed meta-regression-analysis demonstrated that this variation can be partly explained by differences in target disorders (studies of patients suffering from chronic major depression showed greater effects for combined therapies than studies with dysthymic patients), psychotherapy (CBASP yielded greater effects for combined therapies than IPT), and pharmacotherapy (SSRIs decreased the add-on effect of any psychotherapy). Exploratory multiple regression analyses suggested that the most important treatment effect modifier is the use of SSRIs as the pharmacological intervention. The finding of a small add-on effect of psychotherapy on depressive symptoms in the treatment of chronic depression is consistent with previous findings [9], yet we could not confirm that this effect is statistically significant. The lack of statistical significance in this study might be partly due to the smaller amount of studies included in this meta-analysis (which was due to stricter inclusion criteria concerning methodological aspects of the primary studies) and exclusion of an extraordinary positive outliner study. Because the diagnostic subgroup and the type of antidepressant medication was not confounded in our analysis (as it was in the preceding meta-analysis [9]), the assumption that the most relevant effect modifier when comparing combined treatments to pure pharmacological treatments is the type of the antidepressant medication, primarily the use of SSRIs, was strengthened. Even though previous findings showed that studies reporting a lager number of treatment session resulted in lager effect sizes for pure psychotherapeutic interventions [9], no correlation between the number of psychotherapeutic sessions and the superiority of combined treatments could be shown.

Small but statistically significant add-on effects of psychotherapy on the quality of life of chronically depressed patients were demonstrated. No differences in acceptance rates (dropout rates) or long-term effects between combined treatments and pure pharmacological interventions were observed.

### Limitations

Several limitations of the current literature emerged upon this review. First, the sample sizes of primary studies varied greatly and the overall number of studies that were suitable for the systematic review was rather small. Therefore, our analyses were primarily dominated by three large-scale studies [13,26,28]. Leave-one-out sensitivity analyses revealed that single studies had only rather small effects on the results of the meta-analyses, yet the results of the meta-regression-analysis are largely dominated by one study [28] that reported unequivocally positive results for the use of CBASP as add-on psychotherapy to antidepressant medication with nefazodone. Beside the analyses of a number of *a priori* defined treatment effect modifiers we conducted additional explorative meta-regression analyses whose interpretation needs to be considered preliminary. Altogether the number of examined effect moderators was large, particularly in relation to the small number of studies. Therefore we advise careful interpretation of these findings as causal evidence and consider them as observational hypotheses that need further investigation.

Another shortcoming of our review is that the presented evidence is based on clinical trials which focus on high internal validity, yet implementation of combined treatments in routine care is connected to certain challenges. For example patients may perceive practical and emotional barriers prior to psychotherapeutic treatment [33] and the careful management of side effects and interaction effects of complex medication regimes might be more difficult in routine care. To manage both treatments adequately collaborative care models need to be considered as recent research has shown their effectiveness for other forms of depression [34]. Thus, evidence from routine care is needed to conclusively evaluate the add-on effect of psychotherapy.

Third, results of the long-term add-on effect of psychotherapy are preliminary, as only four studies provided relevant data. More research is required because of the high risk of relapse in chronic depression.

## Conclusions

This systematic review could not provide clear evidence for the augmentation of pharmacotherapy with psychotherapy in the treatment of chronic depression. Our findings indicate that the role of add-on psychotherapy may be of more limited value than suggested in current literature [9] and clinical practice guidelines on treatment of chronic depression [10,11]. However, psychotherapy may be a relevant factor for the treatment of chronic depression due to several reasons. First, augmentation of pharmacotherapy with psychotherapy can lead to significant gains in outcomes that are highly relevant for patients, such as quality of life. Second, patients often prefer psychotherapy over medication [35]. Third, as patients’ preference can influence treatment adherence patient’s choice should be considered in the planning of an optimal treatment strategy.

The heterogeneity across studies further highlighted the relevance of the optimization of both psychotherapeutic and pharmacological interventions to increase treatment effects. Our analysis revealed a high impact of SSRIs on the comparative effectiveness of treatment strategies. One explanation for this finding could be that the non-SSRI antidepressants (*e.g.*, moclobemide, nefazodone) are in combination with psychotherapeutic interventions less effective than SSRIs in combination with psychotherapeutic interventions, yet further evidence on the direct comparison of the different types of antidepressants is needed to conclusively interpret this finding. Therefore, in further studies of the combination of pharmacotherapy with psychotherapy SSRIs should be used as a comparator. The heterogeneity across studies further indicated that the category chronic depression subsumes clinically heterogeneous subgroups. Especially patients suffering from dysthymia and chronic major depression seem to respond differently to certain treatment strategies.

Further research on the cost-effectiveness, long-term outcomes and the role of psychotherapy in the continuation and maintenance treatment of chronically depressed patients is required to conclusively assess the value of psychotherapy as an add-on treatment to pharmacotherapy.

## Competing interests

The authors declare that they have no competing interests.

## Authors' contributions

LH, MH, and LK designed the study. AvW, LH, and LK prepared the study protocol. AvW and AW managed the literature searches and the reference database. AvW, LH, AW and LK screened potentially relevant publications. AvW, LH, and LK examined studies for inclusion. AvW and LK performed data extraction. AvW and LK undertook the statistical analyses. All authors contributed to the interpretation of results. AvW wrote the first draft of the manuscript. LH, AW, MH, and LK revised the article critically for important intellectual content. All authors have approved the final manuscript.

## Pre-publication history

The pre-publication history for this paper can be accessed here:

http://www.biomedcentral.com/1471-244X/12/61/prepub
